# Genetic and Functional Analysis of the *DLG4* Gene Encoding the Post-Synaptic Density Protein 95 in Schizophrenia

**DOI:** 10.1371/journal.pone.0015107

**Published:** 2010-12-02

**Authors:** Min-Chih Cheng, Chao-Lin Lu, Sy-Ueng Luu, Ho-Min Tsai, Shih-Hsin Hsu, Tzu-Ting Chen, Chia-Hsiang Chen

**Affiliations:** 1 Department of Psychiatry, Yuli Mental Health Research Center, Yuli Veterans Hospital, Yuli Township, China; 2 Department of Psychiatry, Hualien Armed Forces General Hospital, Xincheng Township, China; 3 Department of Psychiatry, Taoyuan Armed Forces General Hospital, Longtan Township, China; 4 Division of Mental Health and Addiction Medicine, Institute of Population Health Sciences, National Health Research Institutes, Longtan Township, China; 5 Institute of Medical Sciences, Tzu-Chi University, Yuli Township, China; 6 Department of Psychiatry, Chang Gung Memorial Hospital at Linkou and Chang Gung University School of Medicine, Gueishan Township, China; RIKEN Brain Science Institute, Japan

## Abstract

Hypofunction of N-methyl-D-aspartate (NMDA) receptor-mediated signal transduction has been implicated in the pathophysiology of schizophrenia. Post-synaptic density protein 95 (PSD95) plays a critical role in regulating the trafficking and activity of the NMDA receptor and altered expression of the PSD95 has been detected in the post-mortem brain of patients with schizophrenia. The study aimed to examine whether the *DLG4* gene that encodes the PSD95 may confer genetic susceptibility to schizophrenia. We re-sequenced the core promoter, all the exons, and 3′ untranslated regions (UTR) of the *DLG4* gene in 588 Taiwanese schizophrenic patients and conducted an association study with 539 non-psychotic subjects. We did not detect any rare mutations at the protein-coding sequences of the *DLG4* gene associated with schizophrenia. Nevertheless, we identified four polymorphic markers at the core promoter and 5′ UTR and one single nucleotide polymorphism (SNP) at the 3′UTR of the *DLG4* gene in this sample. Genetic analysis showed an association of a haplotype (C–D) derived from 2 polymorphic markers at the core promoter (odds ratio = 1.26, 95% confidence interval = 1.06–1.51, p = 0.01), and a borderline association of the T allele of the rs13331 at 3′UTR with schizophrenia (odds ratio = 1.19, 95% confidence interval = 0.99–1.43, p = 0.06). Further reporter gene assay showed that the C-D-C-C and the T allele of the rs13331 had significant lower activity than their counter parts. Our data indicate that the expression of the *DLG4* gene is subject to regulation by the polymorphic markers at the core promoter region, 5′ and 3′UTR of the gene, and is associated with the susceptibility of schizophrenia.

## Introduction

Compelling evidence suggests that compromised NMDA receptor-mediated signal transduction is implicated in the pathophysiology of schizophrenia [1]. The NMDA receptor hypofunction hypothesis of schizophrenia arises first from the observation that antagonists of the NMDA receptor, such as phencyclidine (PCP) and ketamine induced schizophrenia-like psychosis in normal individuals and exacerbated psychotic symptoms in chronic stable patients with schizophrenia [2]. Further evidence comes from studies reporting that combined use of the co-activators of NMDA receptor, such as D-serine, D-alanine, D-cycloserine, or the glycine transporter 1 inhibitor, such as D-sarcosine, with antipsychotic drugs improves the negative symptoms and cognitive deficits in schizophrenia [3]–[5]. A double-blind study showed that D-sarcosine alone was effective in reducing both positive and negative symptoms in acute schizophrenia patients, especially in drug-naïve patients, which also supports the involvement of reduced NMDA receptor activity in the pathophysiology of schizophrenia [6].

Several postmortem studies of schizophrenia have reported altered expression of NMDA receptor subunits and their interacting molecules in various brain regions of patients with schizophrenia [7]–[16], indicating that dys-regulated expression of the NMDA receptor and its associated molecules may underlie the pathophysiology of schizophrenia [17].

The post-synaptic density protein 95 (PSD95) is a member of the synapse-associated protein family of scaffolding molecules that control the organization, composition, and function of synapses [18]. The PSD95 plays a critical role in regulating NMDA receptor activity and its signal transduction [19]. It binds to the C-terminal of the NMDA receptor subunits NR2A and 2B [20], [21], and controls the trafficking, clustering, and anchoring of NMDA receptors at the postsynaptic membrane [22]. The PSD95 is encoded by the *DLG4* gene. The *Dlg4* knockout mice showed defective synaptic plasticity and impaired spatial learning [23]. Several postmortem studies have revealed altered expression of the PSD95 in various brain regions of patients with schizophrenia [7], [8], [10], [14], [16], [17], [24]. Taken together, these findings indicate that the aberrant expression and function of the PSD95 may contribute to the compromised NMDA receptor-mediated signaling in schizophrenia. Furthermore, the *DLG4* gene that encodes the PSD95 was mapped to chromosome 17p13.1, a region linked to schizophrenia [25]. Thus, the *DLG4* gene is a reasonable candidate gene of schizophrenia in view of the high genetic basis of the etiology of schizophrenia. However, to our knowledge, no mutations of the *DLG4* gene associated with schizophrenia have been identified so far.

The study aimed to investigate whether there are genetic variants of the *DLG4* that may confer an increased risk to schizophrenia. To test this hypothesis, we re-sequenced the core promoter, all the exons, and the 3′ untranslated regions (UTR) of the *DLG4* gene in a sample of Han Taiwanese schizophrenic patients and conducted a case-control association analysis. We also performed reporter gene activity assay to characterize the genetic variants at the 5′ and 3′ends of the *DLG4* gene identified in this study.

## Results

### Detection of Genetic Variants

**Figure 1:**
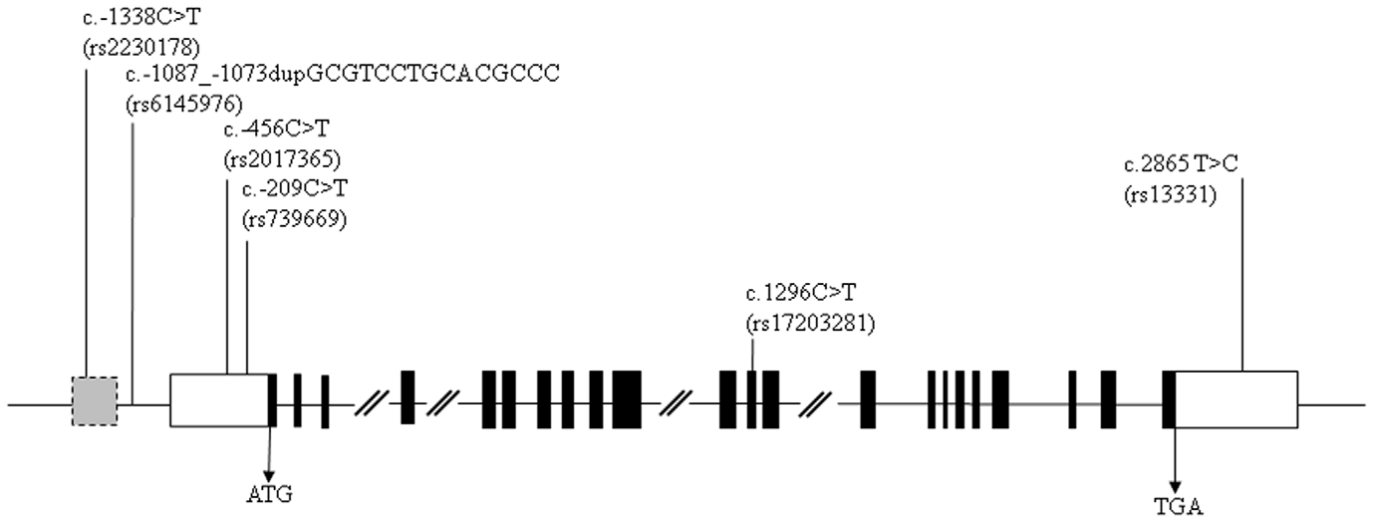
Schematic genomic structure of the *DLG4* gene and locations of six genetic variants identified in this study. The gray box indicates the putative core promoter region; the black box indicates the protein-coding region; the white box indicates the untranslated region.

After sequencing all the protein-coding regions of the *DLG4* gene in our patients, we did not identify any mutations associated with schizophrenia in this sample. Nevertheless, we identified six common variants that have been reported in the single nucleotide polymorphisms (SNP) database (http://www.dbSNP), including rs2230178, rs6145976, rs2017365, rs739669, rs17203281, and rs13331 from 5′ to 3′ of the *DLG4* gene. The rs2230178 and rs6145976 were located at putative core promoter regions, while rs2017365 and rs739669 were located at the untranslated exon 1 of the *DLG4* gene. The rs17203281 was located at exon 12, and the rs13331 was located at the 3′ UTR of exon 22 of the *DLG4* gene. Locations of these variants are illustrated in Figure 1.

### In Silico Analysis

In sillico analysis showed that all the four markers from the 5′end of the *DLG4* gene, i.e. rs2230178, rs6145976, rs2017365 and rs739669 makers, were located at transcription factors binding sites, suggesting these variants may have regulatory influences on the expression of the *DLG4* gene. The rs17203281 was a synonymous mutation that did not change amino acid isoleucine at codon 432. The rs13331 at the 3′UTR was located at a specific translation control element site (differentiation control element, DICE), indicating that it might control the translation of *DLG4*. The putative regulatory element binding sites of these polymorphic markers are listed in Table S1.

### Genetic Association Analysis

**Figure 2:**
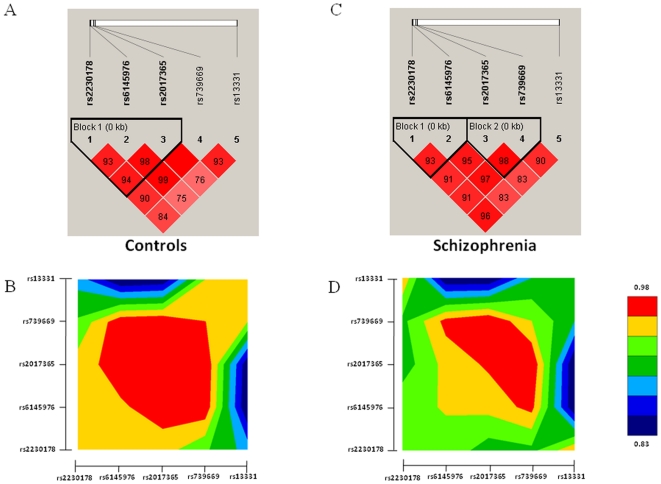
Plots of pair-wise linkage disequilibrium of the five molecular variants of the *DLG4* gene in the patient group and control subjects. (A) and (C) are results using the Haploview computer program, while (B) and (D) are results using the GOLD computer program.

We chose the four polymorphic markers at the 5′ end of the gene (rs2230178, rs6145976, rs2017365, and rs739669) and the marker at the 3′UTR (rs13331) for further genetic association study in view of their potential regulatory function on the expression of *DLG4* gene. The genotype distributions of these markers did not deviate from Hardy-Weinberg equilibrium in both the patient and control groups. Pair-wise linkage disequilibrium analysis showed significant linkage disequilibrium (LD) among these five markers in both patient and control groups. Notably, we found different LD structure between the patient and control groups as shown in Figure 2, suggesting there might be differences in haplotype distributions between patients and control subjects.

Single marker association analysis showed a borderline allelic association of the rs6145976 (p = 0.08), rs739669 (p = 0.04), and rs13331 (p = 0.06) with schizophrenia, as summarized in Table S2. In haplotype-based association analysis, we found a significant association of the haplotype C–D derived from the two polymorphic markers located at the core promoter (rs2230178 and rs6145976, p = 0.01) with schizophrenia, whereas a nominal association of the haplotype C-D-C derived from 3 markers (rs2230178, rs6145976, and rs2017365,p = 0.03), and the haplotype C-D-C-C derived from the four polymorphic markers at the 5′ end of the *DLG4* gene (rs2230178, rs6145976, rs2017365, and rs739669, p = 0.04) with schizophrenia. The haplotype data are listed in Table S3.

### Reporter Gene Activity Assay

**Figure 3:**
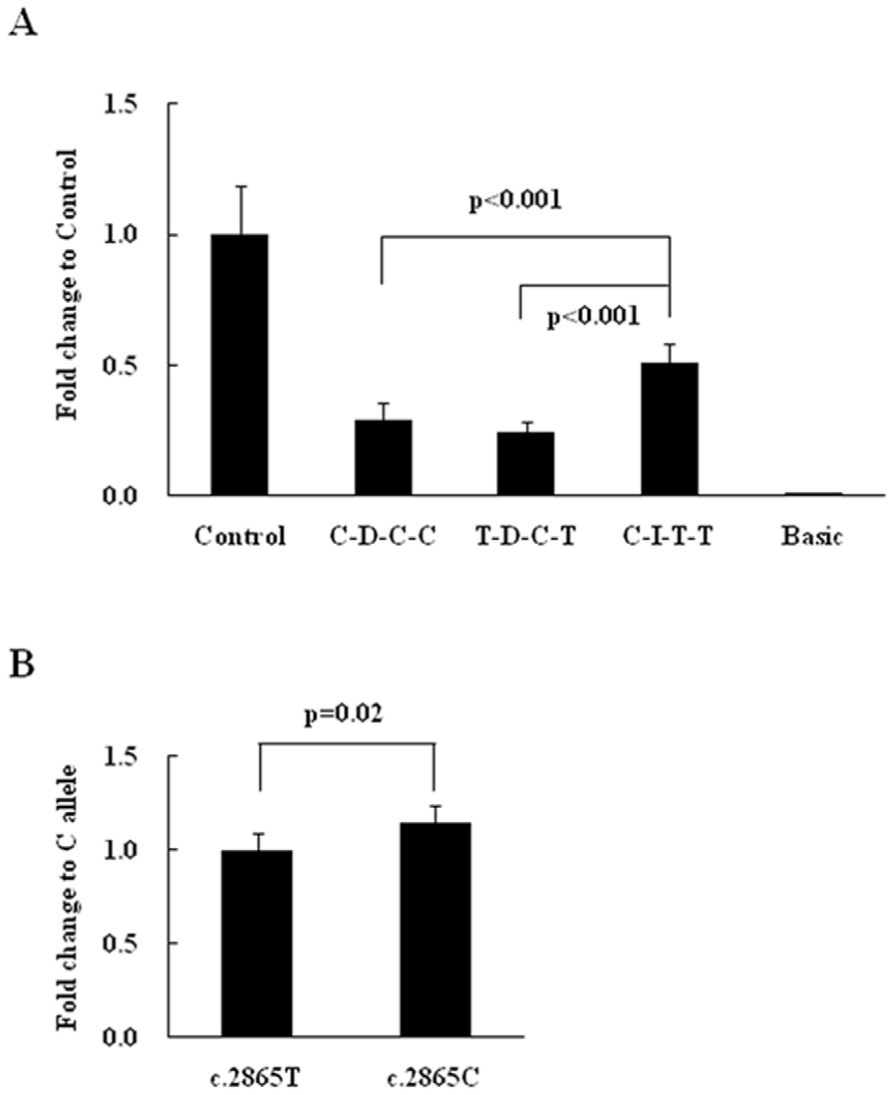
Reporter gene activity assays of molecular variants of the *DLG4* investigated in this study. (A) The promoter activity of the C-D-C-C that is associated with schizophrenia had significantly lower than that of the C-I-T-T haplotype. I: indicates two copies of the “gcgtcctgcacgccc”of the rs6145976; D: indicates single copy of the “gcgtcctgcacgccc” of the rs6145976. (B) The T allele of the rs13331 at 3′UTR that is borderline associated with schizophrenia had significantly lower activity than that of the C allele.

We further performed a reporter gene activity assay to assess the potential regulatory function of these polymorphic variants on the expression of *DLG4* gene. We measured the promoter activity of constructs containing the rs2230178, rs6145976, rs2017365, and rs739669. As shown in Figure 2A, the haplotype C-D-C-C had significantly lower promoter activity compared to the common haplotype C-I-T-T (p<0.001). We also assessed the reporter gene activity of the rs13331 at the 3′UTR of the *DLG4* gene. As shown in Figure 3B, the T allele of the rs1331 had significantly lower activity than the C allele (p = 0.02).

### Power Analysis

The study had the power of 18.4%, 53.0%, and 83.8% to detect the allelic relative risk of 1.1, 1.2, and 1.3, respectively, under the following statistic parameters: multiplicative inheritance mode, disease prevalence = 0.01, risk allele frequency = 0.3, and alpha level of 0.05.

## Discussion

Schizophrenia is a complex genetic disease with polygenic involvement in its etiology. Currently, two hypotheses have been proposed to account for the genetic basis of schizophrenia. One is the common variant hypothesis that attributes schizophrenia to the joint effect of multiple common genetic variants, with each contributing a small-to-modest risk of schizophrenia [26]. The other is the rare mutation hypothesis, which proposes that schizophrenia is caused by rare mutations with a high clinical penetrance, and the pathogenic mutation is highly individualized in each affected patient or family [27]. In this study, after re-sequenced the putative core promoter region, all the exons, and the 3′UTR regions of the *DLG4* gene in our patients, we did not find missense or frameshifting mutations in the *DLG4* gene associated schizophrenia, indicating that exonic mutations in the *DLG4* might be very rare in schizophrenia, and unlikely play a major role in the pathogenesis of schizophrenia.

Nevertheless, we confirmed six polymorphic markers of the *DLG4* gene that have been reported in the SNP database in our subjects, including rs2230178, rs6145976, rs2017365, rs739669, rs17203281, and rs13331. In silico analysis showed that different alleles of rs2230178, rs6145976, rs2017365, and rs739669 at the 5′ end of the gene may have differential influences on expression of the *DLG4* gene. The results of the reporter gene assay support the prediction from in silico analysis; the haplotypes C-D-C-C and T-D-C-T had significant lower activity than the C-I-T-T. The rs17203281 at exon 12 is a synonymous mutation that does not alter the amino acid isoleucine at codon 432. Although several studies have indicated that synonymous mutations may not be always silent [28], [29], we did not pursue the functional significance of this marker in this study, because this marker has been reported not to be associated with schizophrenia in a Han Taiwanese population in a previous study [30]. The rs13331 at the 3′UTR of *DLG4* was also predicted to be functional, because it was located at the DICE site. The reporter gene assay showed C allele had a significantly higher Renilla activity than the T allele, suggesting the rs13331 may also play a role in regulating the expression of *DLG4*.

In the genetic analysis, we found a different LD structure derived from 4 markers at the 5′ end of the gene between the patient and control groups, suggesting that there might be different haplotype distributions between patient and control groups. The different LD might arise from the borderline association of the rs6145976 (p = 0.08) and the rs739669 (p = 0.04) with schizophrenia. Further haplotype-based association analysis showed a significant association of the haplotype C-D derived from two markers at the core promoter region with schizophrenia (rs2230178 and rs6145976, p = 0.01), and reporter gene activity assays showed that the haplotype C-D-C-C derived from rs2230178-rs6145976- rs2017365-rs739669 had significant lower promoter activity than that of the C-I-T-T, suggesting that C-D haplotype that is associated with schizophrenia may have reduced expression of the *DLG4* gene in patients with schizophrenia.

To our knowledge, there are only two genetic association studies of *DLG4* with schizophrenia in the literature. Kawashima and colleagues chose six SNPs (rs314253, rs13331, rs2242449, rs390200, rs507506, rs739669) across the entire *DLG4* gene and examined their association with schizophrenia in a Japanese sample [31]; they found no association of these SNPs and their derived haplotypes with schizophrenia; however, we found a borderline association of the rs13331 SNP with schizophrenia in our sample (p = 0.06). In another study, Tsai and colleagues examined the association of two SNPs (rs2521985 at intron 2 and rs17203281 at exon 12) of the *DLG4* gene with schizophrenia in a Han Taiwanese sample [30]; they found no association of these two SNPs with schizophrenia in their sample.

Based on the genetic and functional analysis of the *DLG4* gene in the present study, we suggest that subjects carrying the C-D haplotype that is associated with schizophrenia and shows a significant low reporter gene activity may have a reduced expression of PSD95. Similarly, subjects that carry the T allele of the rs13331 that is associated with schizophrenia and shows a low reporter gene activity may also have a decreased level of PSD95. Taken together, the present study suggests reduced *DLG4* gene expression may confer increased risk to schizophrenia.

PSD95 plays an essential role in the trafficking, clustering, and anchoring of the NMDA receptor at the postsynaptic membrane [22]. Expression of PSD95 selectively enhances NR2A and NR2B expression, which results in increased NR1/NR2A and NR1/NR2B expression [21]. PSD95 also modulates the channel gating of the NMDA receptor by increasing the channel opening rate [32]. Hence, the reduced PSD95 expression in those subjects carrying the C-D haplotype and the T allele of the rs13331 may lead to a reduction of the functional NMDA receptor or a compromised NMDA receptor-mediated signaling transduction.

Furthermore, PSD95 forms a big protein complex by interacting directly and indirectly with many synaptic adhesion proteins and intracellular molecules [33]. Hence, the influence of reduced expression of the PSD95 may not be limited to the NMDA receptor. For example, PSD95 is required for activity-driven synapse formation [34]; PSD95 also interacts with the dopamine D1 receptor [35] and is involved in the reciprocal facilitating, positive feedback loop between the dopamine D1 receptor and NMDA receptor [36]; PSD95 is also involved in neuroligin-mediated excitatory and inhibitory synapse formation [37]; PSD95 is reported to regulate dendritic spine growth and synaptic plasticity [38]. Taken together, reduced expression of PSD95 may have broad and diverse influences on synaptic plasticity and function. The clinical significance and the relevance of these findings to the pathogenesis and pathophysiology of schizophrenia remain to be explored.

In summary, we characterized a specific haplotype at the promoter and a SNP at the 3′UTR of the *DLG4* gene that were associated with increased liability to schizophrenia. These genetic markers may lead to reduced expression of the PSD95 in the brain, and exert broad and diverse influence on the pathogenesis of schizophrenia. However, the study is limited by its small sample size, and the borderline statistical significance. Independent studies with larger sample size are needed to verify the findings from the present study.

## Materials and Methods

### Subjects

All subjects were Han Taiwanese. Patients fulfilling the diagnostic criteria for schizophrenia as defined by the Diagnostic and Statistical Manual of Mental Disorders-IV (DSM-IV) were recruited into this study from the Department of Psychiatry, Yuli Veterans Hospital, Hualien Armed Forced General Hospital, and Taoyuan Armed Forced General Hospital, Taiwan. The diagnosis of schizophrenia was based on clinical interviews and reviews of medical records by senior psychiatrists with consensus. Exclusion criteria included psychosis due to a general medical condition, substance-related psychosis, and mood disorder with psychotic features. Control subjects were recruited from subjects receiving routine medical checkups from the Department of Family Medicine of a general hospital at eastern Taiwan. The mental status and history of mental illness of the control subjects were evaluated by a senior psychiatrist; subjects diagnosed with a DSM-IV axis I disorder were excluded. The study protocol was approved by the Ethics Committee of Yuli Veterans Hospital, Hualien Armed Forced General Hospital, Taoyuan Armed Forced General Hospital, and Tzu-Chi General Hospital, Taiwan, and written informed consent was obtained after the procedures were fully explained. The patient group comprised 588 schizophrenia patients (333 males, 255 females, mean age = 42.46 years, SD = 11.95), while the comparison group comprised 539 subjects (250 males, 289 females, mean age = 46.00 years, SD = 13.02). Genomic DNA was prepared from peripheral blood using the Puregene DNA purification system (Gentra Systems Inc. Minneapolis, MI), according to the manufacturer's instructions.

### Mutation Detection and Genotyping

The *DLG4* gene (GenBank accession No. NC_000017) contains 22 exons that span approximately 30 kb at chromosome 17p13.1; the exon 1 contains the untranslated region and the translation start site [39]. The putative promoter of the *DLG4* gene was predicted to be located between nucleotide positions –1166 and –1415 upstream from the ATG starting nucleotide using the PROSCAN (http://bimas.dcrt.nih.gov/mdbio). The schematic genomic structure of the *DLG4* gene is illustrated in Figure 1. We systematically screened mutations at the promoter region, all the exons and their flanking intronic sequences of the *DLG4* gene in all patients using PCR-direct autosequencing. Optimal PCR primer sequences were designed to amplify the above regions using Primer3 (http://WWW-genome.wi.mit.edu/cgi-bin/primer/primer3_www.cgi). Primer sequences, optimal annealing temperatures and the size of each amplicon are listed in Table S4. In standard PCR, genomic DNA (100 ng) was amplified in a reaction volume of 20 µl containing 1 µM each of sense and antisense primer, 0.2 mM of dNTP, 50 mM of KCl, 1.5 mM of MgCl_2_, 0.1% vol/vol of Triton X-100, 10 mM of Tris-HCl (pH 9.0), and 2.5 U Taq polymerase. PCR cycling conditions consisted of an initial denaturation at 95°C for 5 min, followed by 30 cycles of 95°C for 1 min, optimal annealing temperature of each amplicon for 1 min, and 72°C for 1 min. PCR was performed with a PTC-200 DNA engine (MJ Research, Watertown, MA). For sequencing, aliquots of PCR products were processed using a PCR Pre-Sequencing Kit (USB Cleveland) to remove residual primers and dNTPs following the manufacturer's protocol. The purified PCR products were subjected to direct sequencing using a ABI Prism^TM^ BigDye^TM^ Terminator Cycle Sequencing Ready Reaction Kit Version 3.1, and a ABI autosequencer 3730 (Perkin Elmer Applied Biosystems, Foster City, USA), according to manufacturer's protocol. For the case-control association study, the genotype of each genetic variant of the *DLG4* gene in the control subjects was also determined by PCR-based direct sequencing.

### In Silico Analysis

The alteration of putative transcription factor binding sites by the promoter variants of the *DLG4* gene were evaluated using TESS (http://www.cbil.upenn.edu/cgi-bin/tess/tess). Putative miRNA targets at the 3′UTR of the *DLG4* gene were analyzed using the program from MicroRNA.org (http://www.microrna.org/microrna/home.do). The potential regulatory elements of the 3′UTR of the *DLG4* gene were assessed using UTRscan (http://www.ba.itb.cnr.it/BIG/UTRScan/).

### Genetic Association Analysis

Deviation from the Hardy-Weinberg equilibrium of the genotype distribution in both the patient and control groups was examined by Chi-square test. Pair-wise linkage disequilibrium analysis of SNPs was performed using Haploview version 4.1 [40] and GOLD-Graphic Overview of Linkage Disequilibrium [41]. Differences in allele, genotype, and estimated haplotype frequencies between patients and controls were evaluated using an online computer platform SHEsis (http://analysis.bio-c.cn) [42]. Post-hoc power analysis was performed using the Genetic Power Calculator (GPC, http://statgen.iop.kcl.ac.uk/gpc/) [43].

### Reporter Gene Activity Assay

Genomic DNAs from the subjects were used for constructing the inserts for the reporter gene assay. For functional characterization of haplotypes derived from rs2230178, rs6145976, rs2017365, and rs739669, sense primer that contains the KpnI recognition site linker (5′-GGTACCTGGCACCAAGAG-3′) and antisense primer that contains the BglII recognition site linker (5′-AGATCTTGCTCCACACAC-3′) were used to PCR amplify the fragment from nucleotides positions –153 to –1441 upstream the ATG starting nucleotide of the *DLG4* gene. The PCR fragments were first cloned into pCR-Blunt II-TOPO vector (Invitrogen, CA, USA) then subcloned into the pGL3-control vector (Promega, Madison, WI, USA), and the authenticity of three haplotypes, designated C-I-T-T, T-D-C-T, and C-D-C-C respectively, were verified by sequencing. Transfection of the plasmids containing each of these three different constructs were performed in a SKNSH neuroblastoma cell line cultured in Minimum Essential Medium (MEM) containing 5% fetal bovine serum in 24-well plates using LipofetamineTM2000 (Invitrogen, California, USA) according to the manufacturer's protocol. Each well contained 10^5^ cells, 800 ng of reporter plasmid, 160 ng of pRL-TK (Promega, Madison, WI, USA) as an internal control, and 2 µl of LipofetamineTM2000. Transfection was repeated 6 times for each reporter plasmid. At 30 hours after transfection, cells were lysed and the luciferase activities were measured using the Dual-Luciferase Reporter Assay System according to the manufacturer's instructions (Promega, Madison, WI, USA). The firefly luciferase activity was normalized against the Renilla luciferase activity in each transfection.

For functional characterization of the c.2865T>C (rs13331), sense primer that contains the XbaI recognition site linker (5′- TCTAGAACACACATTCCAGA-3′) and antisense primer that contains the BamHI recognition site linker (5′-GGATCCTTGGAGTGAAGAAGG- 3′) were used to obtain amplicon containing the rs13331 (c.2865T>C). The amplicon (578 bp) was first cloned into pCR-Blunt II-TOPO vector (Invitrogen, CA, USA) then subcloned into 3′UTR of plasmid pRL-TK (Promega, Madison, WI, USA). The authenticity of these clones was verified by sequencing, and designated c.2865T and c.2865C, respectively. Transfection was performed in SKNSH neuroblastoma cell line cultured in Minimum Essential Medium (MEM) containing 5% fetal bovine serum in 24-well plates. Each well contained 10^5^ cells, 1 µg of reporter plasmid, 200 ng of pGL3-Control as an internal control reporter, and 2 µl of LipofetamineTM2000. Every treatment was repeated six times. At 30 hours after transfection, cells were lysed and the luciferase activities were measured using the Dual-Luciferase Reporter Assay System, according to the manufacturer's instruction. The Renilla luciferase activity was normalized to the firefly luciferase activity, and reported as the relative luciferase ratio. The differences in gene expression activity between the two constructs were analyzed using the t-test, while differences among the three groups were evaluated by analysis of variance (ANOVA) followed by post-hoc comparison; the p value was set at 0.05.

## Supporting Information

Table S1 **In silico analysis of variants of the** ***DLG4*** **gene identified in this study.**
(DOC)

Table S2 **Genetic association data of the** ***DLG4*** **gene in this study.**
(DOC)

Table S3 **Haplotype-based association study of the** ***DLG4*** **gene and schizophrenia.**
(DOC)

Table S4 **Primers sequences for PCR amplification of the putative core promoter, all the exons, and 3**′**UTR of the** ***DLG4*** **gene, and optimal annealing temperature, and sizes of PCR products.**
(DOC)
