# Correction: Genetic and Functional Analysis of the *DLG4* Gene Encoding the Post-Synaptic Density Protein 95 in Schizophrenia

**DOI:** 10.1371/annotation/8e156c1b-2369-45da-93f7-99701f5935d2

**Published:** 2010-12-30

**Authors:** Min-Chih Cheng, Chao-Lin Lu, Sy-Ueng Luu, Ho-Min Tsai, Shih-Hsin Hsu, Tzu-Ting Chen, Chia-Hsiang Chen

In all affiliations, China should be changed to Taiwan.

