# Supplementary material for: Genetic and Functional Analysis of the DLG4 Gene Encoding the Post-Synaptic Density Protein 95 in Schizophrenia
Source: PLoS One. 2010 Dec 2;5(12):e15107. doi: 10.1371/journal.pone.0015107 (PMC2996301; doi:10.1371/journal.pone.0015107)
Supplement: Table S1 — In silico analysis of variants of the DLG4 gene identified in this study. (DOC) [file pone.0015107.s001.doc]

# Table S1. In silico analysis of variants of the *DLG4* gene identified in this study

| dbSNP ID | Allele locations | Putative regulatory element binding |
| --- | --- | --- |
| rs2230178 | c.-1338C>T | C allele: gammaCAC1; gammaCAC2; CACCC-binding factor; AP-2 factor  T allele: GCR1 factor |
| rs6145976 | c.-1087_-1073dup  GCGTCCTGCACGCCC | Duplication allele: Yi factor; Ttk factor; LVc factor; MBF-1 factor; MTF-1 factor; SP-1 factor; NF-1; T-Ag; USF  Non-duplication allele: Opaque-2; TGA1a, 1b; TAF-1; CPRF-2; CPRF-3; AP-1; ATF-1; LRF-1; ASF-1; MSN4; deltaCREB; HBP-1; TREB-1; ATF-1; ATF; c-Jun; CREB; CRE-BP1; TREB-1; EivF; ATF3; HBP-1, bZIP910 |
| rs2017365 | c.-456C>T | C allele: DEP2 factor; YY1 factor; muEBP factor; twi factor; TFE3-S factor; muEBP-C2 factor; TFE3-S factor; USF1 factor |
| rs739669  rs17203281 | c.-209C>T  c.1296C>T | C allele: GAGA factor  Silent mutation (Ile432) |
| rs13331 | c.2865T>C | T allele: Differentiation control element (DICE) |
