# Supplementary material for: Genetic and Functional Analysis of the DLG4 Gene Encoding the Post-Synaptic Density Protein 95 in Schizophrenia
Source: PLoS One. 2010 Dec 2;5(12):e15107. doi: 10.1371/journal.pone.0015107 (PMC2996301; doi:10.1371/journal.pone.0015107)
Supplement: Table S2 — Genetic association data of the DLG4 gene in this study. (DOC) [file pone.0015107.s002.doc]

**Table S2.** Genetic association data of the *DLG4* gene in this study.

| Variant | Population | Sample | Genotype | | | HWE p | p | Allele | | p |
| --- | --- | --- | --- | --- | --- | --- | --- | --- | --- | --- |
| rs2230178  (c.-1338C>T) |  |  | C/C | C/T | T/T |  |  | C | T |  |
|  | Schizophrenia | 588 | 430 (73.1%) | 146 (24.8%) | 12 (2.0%) | 0.92 | 0.60 | 1006 (85.5%) | 170 (14.5%) | 0.35 |
|  | Control | 539 | 383 (71.1%) | 141 (26.2%) | 15 (2.8%) | 0.64 |  | 907 (84.1%) | 134 (15.9%) |  |
| (rs6145976)  c.-1087_-1073  dupGCGTCCTGCACGCCC |  |  | I/I | I/D | D/D |  |  | I | D |  |
|  | Schizophrenia | 573 | 137 (23.9%) | 291 (50.8%) | 145 (25.3%) | 0.70 | 0.12 | 565 (49.3%) | 581(50.7%) | 0.08 |
|  | Control | 537 | 158 (29.4%) | 254 (47.3%) | 125 (23.3%) | 0.24 |  | 570 (53.1%) | 504 (46.9%) |  |
| rs2017365  (c.-456C>T) |  |  | C/C | C/T | T/T |  |  | C | T |  |
|  | Schizophrenia | 517 | 126 (24.4%) | 263 (50.9%) | 128 (24.8%) | 0.69 | 0.34 | 515 (49.8%) | 519 (50.2%) | 0.20 |
|  | Control | 518 | 118 (22.8%) | 251 (48.5%) | 149 (28.8%) | 0.53 |  | 487 (47.0%) | 549 (53.0%) |  |
| rs739669  (c.-209C>T) |  |  | C/C | C/T | T/T |  |  | C | T |  |
|  | Schizophrenia | 524 | 61 (11.6%) | 246 (47.0%) | 217 (41.4%) | 0.49 | 0.10 | 368 (35.1%) | 680 (64.9%) | 0.04 |
|  | Control | 523 | 52 (9.9%) | 220 (42.1%) | 251 (48.0%) | 0.71 |  | 324 (31.0%) | 722 (69.0%) |  |
| (rs13331)  c.2865T>C |  |  | T/T | T/C | C/C |  |  | T | C |  |
|  | Schizophrenia | 528 | 70 (13.2%) | 265 (50.2%) | 193 (36.6%) | 0.16 | 0.08 | 405 (38.4%) | 651 (61.6%) | 0.06 |
|  | Control | 512 | 62 (12.1%) | 228 (44.5%) | 222 (43.4%) | 0.77 |  | 352 (34.4%) | 672 (65.6%) |  |

D: one copy of the GCGTCCTGCACGCCC; I: two copies of the GCGTCCTGCACGCCC; HWE: Hardy-Weinberg equilibrium
