# Supplementary material for: Genetic and Functional Analysis of the DLG4 Gene Encoding the Post-Synaptic Density Protein 95 in Schizophrenia
Source: PLoS One. 2010 Dec 2;5(12):e15107. doi: 10.1371/journal.pone.0015107 (PMC2996301; doi:10.1371/journal.pone.0015107)
Supplement: Table S3 — Haplotype-based association study of the DLG4 gene and schizophrenia. (DOC) [file pone.0015107.s003.doc]

**Table S3.** Haplotype-based association study of the *DLG4* gene and schizophrenia.

| Haplotype | rs2230178 | rs6145976 | rs2017365 | rs739669 | Frequency (%) | | Pearson’s p | Odds ratio (95% CI) | Global P |
| --- | --- | --- | --- | --- | --- | --- | --- | --- | --- |
|  |  |  |  |  | Case | Controls |  |  |  |
| 2 SNPs |  |  |  |  |  |  |  |  | 0.04* |
| 1 | C | Del | - | - | 36.5 | 31.3 | 0.01* | 1.26 (1.06-1.51) |  |
| 2 | C | Ins | - | - | 49.0 | 52.8 | 0.07 | 0.86 (0.72-1.01) |  |
| 3 | T | Del | - | - | 14.2 | 15.5 | 0.38 | 0.90 (0.71-1.14) |  |
| 3 SNPs |  |  |  |  |  |  |  |  | 0.09 |
| 1 | C | Del | C | - | 35.5 | 31.4 | 0.03* | 1.24 (1.02-1.49) |  |
| 2 | C | Ins | T | - | 48.8 | 52.5 | 0.21 | 0.89 (0.75-1.07) |  |
| 3 | T | Del | C | - | 13.1 | 15.2 | 0.22 | 0.85 (0.67-1.10) |  |
| 3 SNPs |  |  |  |  |  |  |  |  | 0.17 |
| 1 | - | Del | C | C | 34.4 | 31.1 | 0.06 | 1.20 (0.99-1.44) |  |
| 2 | - | Del | C | T | 14.0 | 15.6 | 0.41 | 0.90 (0.71-1.15) |  |
| 3 | - | Ins | T | T | 49.0 | 52.5 | 0.24 | 0.90 (0.75-1.07) |  |
| 4 SNPs |  |  |  |  |  |  |  |  | 0.12 |
| 1 | C | Ins | T | T | 48.7 | 52.3 | 0.19 | 0.89 (0.74-1.06) |  |
| 2 | C | Del | C | C | 34.2 | 30.5 | 0.04* | 1.22 (1.01-1.47) |  |
| 3 | T | Del | C | T | 12.8 | 14.4 | 0.37 | 0.89 (0.69-1.15) |  |

Ins: Insertion; Del: non-insertion; *: p<0.05
