# Supplementary material for: Genetic and Functional Analysis of the DLG4 Gene Encoding the Post-Synaptic Density Protein 95 in Schizophrenia
Source: PLoS One. 2010 Dec 2;5(12):e15107. doi: 10.1371/journal.pone.0015107 (PMC2996301; doi:10.1371/journal.pone.0015107)
Supplement: Table S4 — Primers sequences for PCR amplification of the putative core promoter, all the exons, and 3′UTR of the DLG4 gene, and optimal annealing temperature, and sizes of PCR products. (DOC) [file pone.0015107.s004.doc]

# Table S4. Primers sequences for PCR amplification of the putative core promoter, all the exons, and 3’UTR of the *DLG4* gene, and optimal annealing temperature, and sizes of PCR products

| Primer | Forward | Reverse | Ta (℃) | Size (bp) |
| --- | --- | --- | --- | --- |
| Promoter | 5’-AACACCTGATCTGTGGTGAGCT-3’ | 5’-GTGGACGATGAGTCAGGGTTAG-3’ | 60 | 777 |
| Exon 1 | 5’-TCACTGCCCCTCCCTTAGTA-3’ | 5’-GGAAAGATTTGGGGAGAAGG-3’ | 62 | 642 |
| Exon 2-3 | 5’-TCTGGGGAGATGAAGTGACA-3’ | 5’-GACTCAGGCTTTTGTCAGCA-3’ | 62 | 597 |
| Exon 4 | 5’-GCTGTTGGGAGAGTGGAGAG-3’ | 5’-CTACCTTGAAGGGGGAGAGG-3’ | 62 | 350 |
| Exon 5-6 | 5’-GCTTGGCTAAGAGGCTGAAA-3’ | 5’-AGACCACATGGCAGAAAGA-3’ | 62 | 422 |
| Exon 7-8 | 5’-TCTTTCTGCCATGTGGTCTG-3’ | 5’-AGCCAAGACCTGGATGGAG-3’ | 62 | 637 |
| Exon 9-10 | 5’-CATCGTTCGCCTCTATGTCA-3’ | 5’-GAGGGACAGCTACAGGGATG-3’ | 64 | 691 |
| Exon 11 | 5’-ACCCTGGGGAGAAGGTTG-3’ | 5’-GCTCATGGCTGGCATTTC-3’ | 62 | 587 |
| Exon 12-13 | 5’-GACCAGATCCTGTCGGTGAG-3’ | 5’-TGTTAACCTGTGCAGCCAAG-3’ | 65 | 717 |
| Exon 14 | 5’-CGGGTCTTTGGGTGACTG-3’ | 5’-GGAGTTCAGAGGGCAAACC-3’ | 62 | 355 |
| Exon 15-18 | 5’-TGGCCCAGAGTATGGAGAAGGG-3’ | 5’-ATCTCCCTACACACCGATCCCC-3’ | 65 | 731 |
| Exon 19 | 5’-GGGGATTTGATTGAAGTAGGC-3’ | 5’-AGGAGAAGGAGGTGGAGGAG-3’ | 62 | 393 |
| Exon 20 | 5’- ACTTCCCATTCCCCACCTCT-3’ | 5’-AAGGGAGAGTTGAACGCAGA-3’ | 60 | 300 |
| Exon 21 | 5’-TGCTATGTGCTCCAAATAGAAGA-3’ | 5’-GCACCTCTTTCTAAGCCATCC-3’ | 62 | 299 |
| Exon 22 | 5’-CCAGGACTTATGGCTTGGAG-3’ | 5’-TCTGGAATGTGTGTGGGAGA-3’ | 60 | 577 |
| 3`UTR-1 | 5’-CTCTCAGGCCCCTACATCTG-3’ | 5’-GGAGTCCCCTGCTTCTCC-3’ | 60 | 582 |
| 3`UTR-2 | 5’-TCTCCCACACACATTCCAGA-3’ | 5’-CTGGCTTGGAGTGAAGAAGG-3’ | 60 | 575 |
